# Supplementary material for: “We need our own clinics”: Adolescents’ living with HIV recommendations for a responsive health system
Source: PLoS One. 2021 Jul 1;16(7):e0253984. doi: 10.1371/journal.pone.0253984 (PMC8248739; doi:10.1371/journal.pone.0253984)
Supplement: S1 File — (DOCX) [file pone.0253984.s001.docx]

**In-depth interview guide**

| Protocol Title: | A description of the mental health outcomes of HIV positive adolescents accessing care in Johannesburg |
| --- | --- |
| Principal Investigator: | Nataly Woollett |

***NOTE: This interview will be recorded, ensure consent given for recording***

**INTRODUCTION**

Thank you for agreeing to talk with me today.

The questions I have don’t have any right or wrong answers. I am interested in any experiences, stories, and ideas you’d like to share. Please feel free to share your honest thoughts and opinions. I would like to ask you to remember that what is said here today is confidential (which means that I can’t talk to others about what we talk about and if I reference your opinion anywhere outside of our conversation your name would never be used).

Do you have any questions for me before we begin?

Great. Let’s get started.

**IN DEPTH INTERVIEW DISCUSSION GUIDE**

Adolescents who are HIV positive and need to attend clinics for their health have lots of good ideas for healthcare providers on what they need from the healthcare system. Adolescents may require more help than just getting ARVs and I would like to ask you today for your opinion on these issues. Some HIV positive adolescents have had difficult experiences growing up; I would also like to ask you about these sorts of experiences too. Especially important is to get your perspective on what the risks are that would make adolescents feel bad and what the resilience (emotional strengthen) factors are for adolescents to be okay.

**Risk factors**

- In your opinion, what are some of the challenges faced by HIV positive adolescents?
- Do you think these challenges would be different if you were born with HIV or if you got infected later? Why?
- In your opinion, what kinds of mental health problems do HIV positive adolescents face? (A mental health problem is something like depression, trauma, alcohol abuse etc.)
- Do you think these mental health problems are different in HIV positive adolescents as opposed to HIV negative adolescents? How come?
- What do you think some of the challenges are for HIV positive adolescents to take care of themselves?
- Do you think HIV positive adolescents experience difficulties in their relationships with family members, friends and romantic partners? What are some of these difficulties?
- What do you think HIV positive adolescents need from others for life to turn out well?
- What behaviours normally show that people are having a hard time managing their feelings?
- What have been some of the more difficult things you have had to deal with in your life until now? (So far, what is the most difficult thing that you have had to cope with in your life?)

**Resilience factors**

- Are there people in your life you can go to when life gets tough? Who are these people and what do they offer you?
- What do HIV positive adolescents need from the healthcare system (by healthcare system I mean hospitals, clinics, or any health facility one goes to when sick)?
- How can the healthcare system be improved to help adolescents more?
- For adolescents who have a great deal of difficulty that they have managed (e.g. parent dies, get separated from siblings, get very sick, school is tough, people discriminate against them because of their status etc.) what helps them cope and come out of the situation okay?
- What sorts of things help you manage life and its stress?
- Who do you look up to and why?
- If you were president and had a million rand, what would you do for adolescents in your situation?

Is there anything else you would like to tell me about the topics we have discussed today?

If not, that is the end of our discussion. Do you have any questions for me?

Thank you for your time and participation, it is really appreciated!
