# Supplementary material for: “We need our own clinics”: Adolescents’ living with HIV recommendations for a responsive health system
Source: PLoS One. 2021 Jul 1;16(7):e0253984. doi: 10.1371/journal.pone.0253984 (PMC8248739; doi:10.1371/journal.pone.0253984)
Supplement: Attachment — Submitted filename: PLosOne reviewer response_April 2021.docx [file pone.0253984.s003.docx]

29^th^ April, 2021

Dear Dr. Zanoni and PLoS One Reviewers,

We thank the reviewers for their suggested edits to our manuscript entitled ***“We need our own clinics”: HIV infected adolescents’ recommendations for a responsive health system***

(Manuscript ID PONE-D-20-34639).

We appreciate the time taken by the reviewers to provide detailed feedback on our manuscript and hope we have sufficiently responded to the next round of feedback.

Sincerely,

Nataly Woollett

Reviewer #3: This is an important study given the increasing numbers of adolescents living with HIV surviving into adulthood and eventual need to transition to adult-oriented care settings. The authors attempt to address this critical time by examining the attitudes and experiences of perinatally infected adolescents living with HIV regarding transition from pediatric to adult care in South Africa, including their recommendations for a successful transition. The study provided insights from adolescents about this critical time through in-depth interviews, including the importance of clinic-based support groups, the value of lay counselors in providing education, counseling, and support to adolescents, increasing widespread education of different modes of HIV transition (especially vertical transmission), and linking the clinic to the community.

The authors have adequately responded to the previous reviewers’ comments; This is my first review of this revised manuscript. Overall, the methods, interpretation, and communication of the findings are excellent. The major strengths of this manuscript are the focus on a population (perinatally-infected adolescents living with HIV in South Africa) that has high rates of morbidity and mortality at each point of the continuum of care, especially healthcare transition, as well as a richness of quotations from the participants to support your discussion.

*Thank you Reviewer 3 for that positive feedback.*

However, a few additional points to consider, including the use of identity-first vs person-first language, unclear methodology with regards to intercoder agreement, incorrect formatting of table 1, and lack of synthesis of data to broader analytical themes.

1. I am concerned with the authors use of identity-first language in the title and throughout the manuscript. The authors should use person-first language in the title and throughout the manuscript. For example: “HIV infected adolescents” can be changed to “adolescents living with HIV;” and “HIV infected women” can be changed to “women living with HIV.”

*Thank you for bringing this important point to our attention. Please note that person-first language has now been incorporated throughout the text.*

2. Additional concern with the methodology is regarding the intercoder reliability; specifically how were discrepancies between independent coders resolved?

*A group of 3 researchers where part of the analysis team and we double coded every transcript. We met weekly to discuss the coding, resolve any conflicts and learn from the data in an iterative way. We were not aiming for perfect alignment in terms of intercoder reliability but rather consensus in terms of the meaning of codes and we felt we were able to reach this successfully through this process. We have added the following to improve clarity:*

To ensure intercoder consensus, fine codes were developed by three researchers experienced in qualitative data analysis by printing out a full set of excerpts (from each data set) associated with each code for each transcript and identifying sub-themes arising from the data. Two researchers applied the thematic codes to every transcript. Each week during the double coding, the findings were critiqued and discussed within the group to guarantee research results. During analysis, we found that data reached a point of saturation, which suggests the sample size was sufficient.

3. Within the results section, table 1 is formatted incorrectly with the table split with text dividing the table. Authors should fix the formatting for table 1.

*Apologies, the table has been realigned now. Thanks.*

4. An additional concern with the results section is the authors dependence on quotations and their interpretation of these quotations for development of descriptive themes. The authors could consider synthesizing results to a higher order to generate new analytical themes or explanations.

*Although higher order analysis may strengthen this manuscript, we do believe we were able to use a grounded theory approach to analysis effectively. We were interested in analyzing the data and deriving meaning from it in the first instance. We feel through this method we were able to meaningfully construct and describe the views and lives of our participants, adding to the literature around their recommendations for their healthcare and bringing their voices more demonstrably to that conversation.*
